# Pulmonary Embolism in COVID-19 Pneumonia: Random Association or Causality?

**DOI:** 10.7759/cureus.8900

**Published:** 2020-06-29

**Authors:** Ked Fortuzi, Haider Ghazanfar, Asim Haider, Komal Patel, Madanmohan Patel

**Affiliations:** 1 Internal Medicine, Bronxcare Health System, Bronx, USA

**Keywords:** covid-19, pulmonary embolism (pe), anticoagulant therapy, epidemic, mortality

## Abstract

Coronavirus disease 2019 (COVID-19) is an ongoing pandemic. COVID-19 leads to a plethora of clinical syndromes, most commonly affecting the pulmonary system but also the cardiovascular, hematologic, and gastrointestinal systems. There is emerging evidence of an association between COVID-19 and venous thromboembolism (VTE). In this report, we have discussed three cases with a COVID-19 infection, whose clinical course was complicated by the presence of pulmonary embolism (PE) without evident risk factors for VTE. All three patients presented with hypoxia and were found to have elevated D-dimer levels. Subsequently, the patients underwent computed tomography (CT) angiography of the chest, which confirmed the presence of pulmonary embolism. Anticoagulation was initiated per guidelines. There is a need to have a low threshold for suspecting pulmonary embolism in patients with COVID-19 infection who present with a sudden onset of severe hypoxia. There is a dire need to increase awareness among health care providers regarding this manifestation of the virus.

## Introduction

COVID-19 arose in China in late November 2019 and quickly developed into a worldwide pandemic. Coronavirus disease 2019 (COVID-19) is caused by severe acute respiratory syndrome coronavirus 2 (SARS-CoV-2). Though most patients affected with this novel virus have mild disease, some develop a more severe affliction that requires hospitalization. The most common reason for hospitalization from COVID-19 infection is respiratory distress [1]. Those with severe disease could rapidly deteriorate into acute respiratory distress syndrome, sepsis, septic shock, and sudden death.

As more information is available, it appears that COVID-19 not only causes respiratory disease, but it also seems to cause systemic disease. This systemic disease has been associated with gastrointestinal, hematologic, and cardiac problems [2]. There is a documented association between this infection and an increased pro-inflammatory state [3]. The viral infection has been associated with elevated inflammatory markers such as lactate dehydrogenase, ferritin, C-reactive protein, D-dimer, and interleukin levels. There has been evidence to suggest that there is a hypercoagulable state associated with this condition, which can manifest as microthrombi or thromboembolic disease [4]. There are emerging data suggesting an association between COVID-19 and venous thromboembolism [5-7], and a high correlation of increasing D-dimer levels and the manifestation of VTE. Computed tomography (CT) angiography remains the primary imaging choice to diagnose this condition. We are presenting three cases of PE in patients with COVID-19 without significant previous risk factors for VTE, who were treated in our facility amidst the epicenter of the COVID-19 pandemic in New York City.

## Case presentation

Case number one

The patient is a 52-year-old male, with a past medical history significant for morbid obesity (with body mass index (BMI) 35), who presented in the emergency department with complaints of fatigue, fever, and cough for five days. On arrival, the patient was hypoxic, saturating 82% on room air, which improved to 94% on oxygen supplementation via nasal cannula. The patient’s chest X-ray showed bilateral interstitial infiltrates. The patient’s initial labs showed a white blood cell (WBC) count of 10.2, lactate dehydrogenase (LDH) 687 units/mL, C-reactive protein (CRP) 454 mg/L, prothrombin (PT) 13.8/partial thromboplastin time (aPTT) 30.9, ferritin 673 ng/mL, procalcitonin 0.48 ng/mL, and elevated D-dimer of 56,698 ng/mL. The diagnosis of COVID-19 was confirmed by the direct detection of SARS-COVD-2 ribonucleic acid (RNA) by the nucleic acid amplification test, primarily reverse transcription-polymerase chain reaction (RT-PCR).

**Figure 1:**
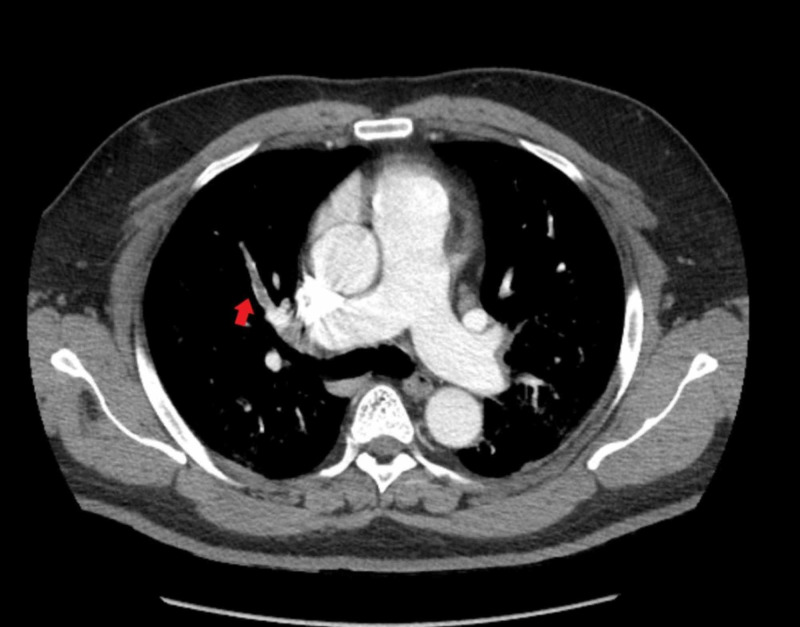
Computerized tomography angiography of the chest showing pulmonary embolism in the right upper lobe anteriorly along with bilateral profuse ground-glass opacities

**Figure 2:**
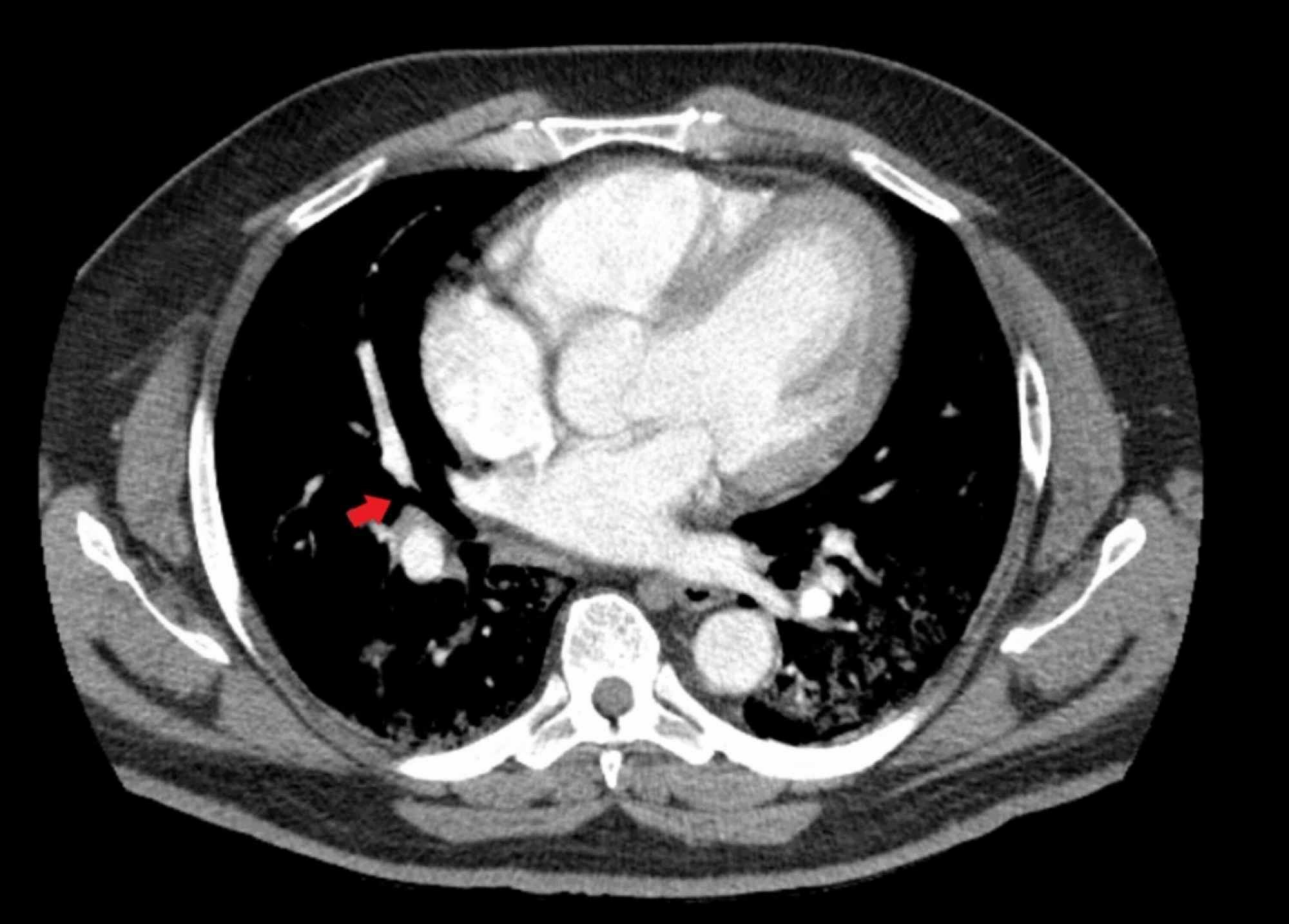
Computerized tomography angiography of the chest showing pulmonary embolism in the right middle lobe along with bilateral profuse ground-glass opacities

The patient denied having a family history of the hypercoagulable disease, recent surgery, trauma, immobilization, cancer, or cigarette smoking. The patient was suspected of having pulmonary embolism because of hypoxia, tachypnea, tachycardia, and elevated D-dimer. Apart from morbid obesity, no other risk factors for pulmonary embolism were identified. The patient underwent computerized tomography (CT) angiography of the chest, which showed PE in the right upper lobe anteriorly and right middle lobe, along with bilateral profuse ground-glass opacities (Figure 1 and Figure 2).

Subsequently, the patient was initiated on intravenous heparin infusion for the pulmonary embolisms. During admission, the patient also received hydroxychloroquine, azithromycin, amoxicillin-clavulanate, and oseltamivir. The patient continued to deteriorate and required intubation and was transferred to the intensive care unit for further management. Furthermore, the patient’s clinical course was complicated by the development of acute tubular necrosis and persistent hyperkalemia. The patient then developed septic shock, which eventually led to the patient’s demise.

Case number two

The patient is a 74-year-old female, with a past medical history of neurofibromatosis and hypertension, who presented with complaints of worsening dry cough, diarrhea, nausea, malaise, body aches, fever, and shortness of breath for a total of two weeks prior to presentation. The patient was noted to be hypoxic on arrival to the emergency department, and requirements for oxygen supplementation were increasing (6L via nasal cannula). Laboratory tests showed WBCs 13.1, LDH 737 units/mL, CRP 80.7 mg/L, elevated D-dimer of 5021 ng/mL, ferritin 310.4 ng/mL, and procalcitonin 0.01 ng/mL. COVID-19 was confirmed by the direct detection of SARS-CoV-2 RNA by nucleic acid amplification test, primarily reverse RT-PCR.

The patient was initially started on hydroxychloroquine, azithromycin, amoxicillin-clavulanate, and oseltamivir. Hydroxychloroquine and azithromycin were stopped due to the development of a prolonged QTc. Due to deterioration in clinical status, the patient’s antibiotics were changed to doxycycline, vancomycin, and cefpodoxime.

**Figure 3:**
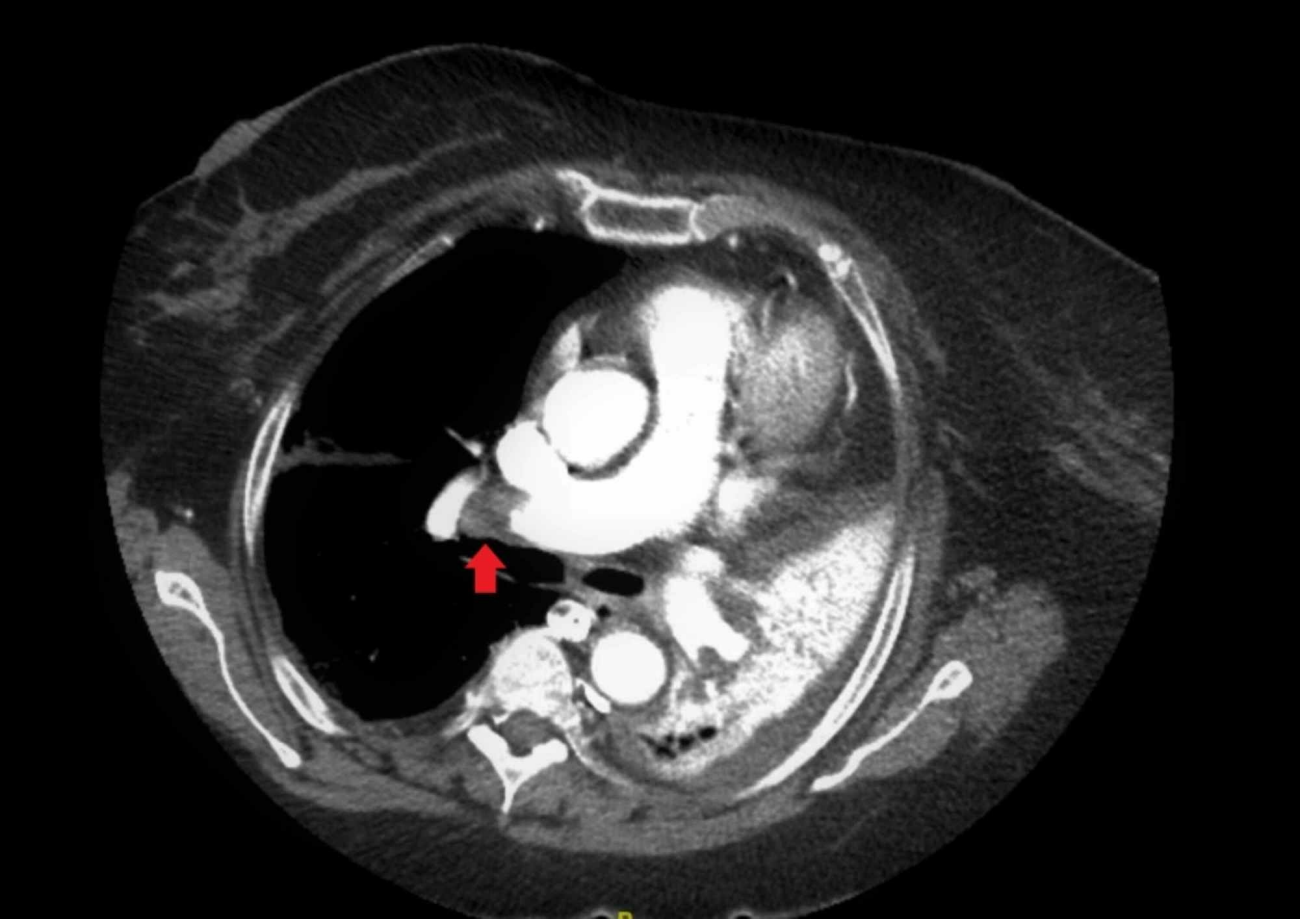
Computerized tomography angiography of the chest showing bilateral pulmonary emboli within the main pulmonary arteries and lower lobe pulmonary arteries

**Figure 4:**
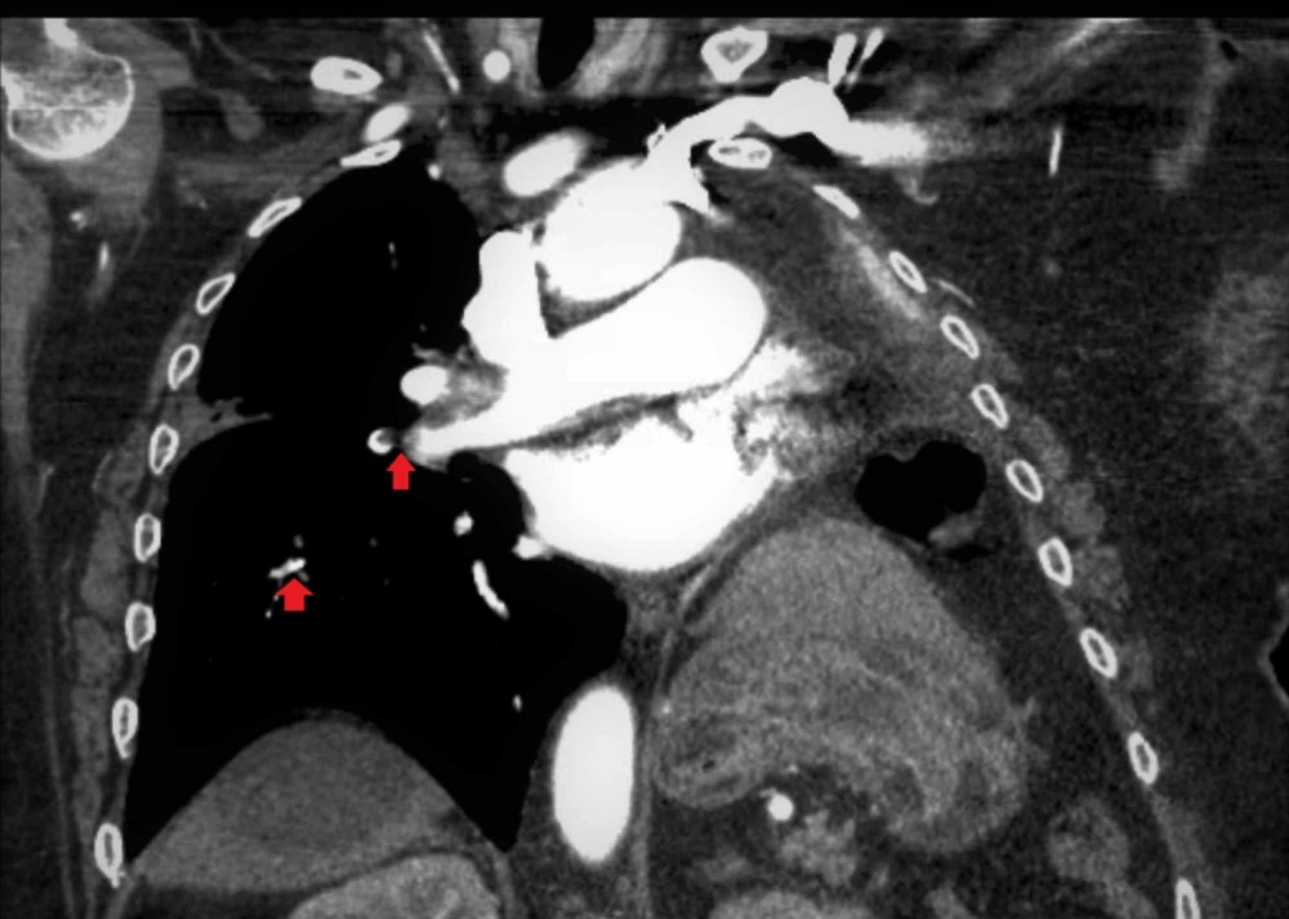
Computerized tomography angiography of the chest showing bilateral pulmonary emboli within the main pulmonary arteries and lower lobe pulmonary arteries (coronal view)

The patient’s hypoxia continued to worsen. On Day 6 of the admission, due to the initial elevated D-dimer level, the patient underwent CT angiography of the chest. The exam showed bilateral pulmonary emboli within the main pulmonary arteries and lower lobe pulmonary arteries (Figures 3-4).

The patient was then started on enoxaparin 1 mg/kg every 12 hours for the therapeutic range. During this time, the patient had remained hemodynamically stable. CT did not show any evidence of right heart strain. The patient’s clinical condition continued to improve. Eventually, the patient was successfully discharged home on apixaban.

Case number three

The patient is a 31-year-old male, with a past medical history of asthma, who presented initially with cough, malaise, and fever, for which he was hospitalized for three days with a diagnosis of COVID-19. The patient was eventually discharged after symptoms had resolved. Three weeks after the initial presentation, the patient returned to the hospital with complaints of shortness of breath and chest pain. The patient denied a family history of a hypercoagulable disease, recent surgery, trauma, immobilization, cancer, or cigarette smoking.

Laboratory tests showed WBC 13.8, LDH 203 units/mL, CRP 453 mg/L, elevated D-dimer of 2134 ng/mL, ferritin 1219 ng/mL. The patient’s initial laboratory findings during the first admission showed an elevated D-dimer of 2134 ng/mL. COVID-19 was confirmed by direct detection of SARS-CoV-2 RNA by nucleic acid amplification test, primarily RT-PCR.

**Figure 5:**
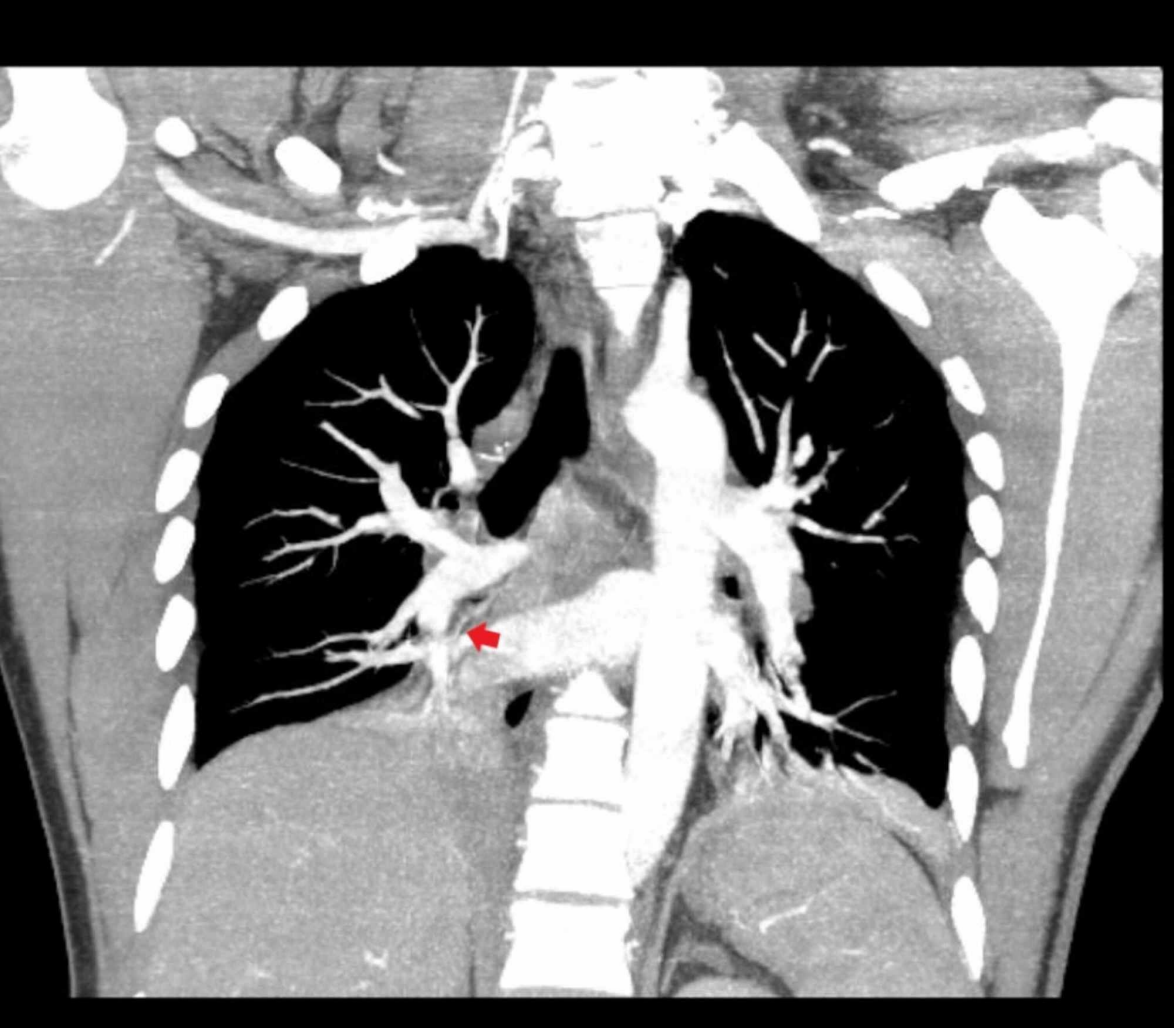
Computerized tomography angiography of the chest showed evidence of subsegmental pulmonary embolism in the right lower lobe

The patient did undergo CT angiography of the chest did not show evidence of thromboembolism during initial admission. During the subsequent admission when the patient developed chest pain and hypoxia, CT angiography of the chest was repeated. The new CT angiography of the chest showed evidence of subsegmental pulmonary embolism on the right lower lobe (Figure 5).

A lower extremity Doppler scan was done, and it was found to be negative for deep vein thrombosis. During the admission, the patient received doxycycline and amoxicillin-clavulanate. The patient was also initiated on apixaban for the management of acute pulmonary embolism. The patient is currently responding to treatment and hypoxia has improved. The patient is planned for discharge home.

The patient characteristics and laboratory findings of all three cases have been mentioned in Table 1.

**Table 1:** Patient’s characteristics and laboratory findings CT: Computed Tomography; WBC: White Blood Cell; ALT: Alanine Transaminase; AST: Aspartate Aminotransferase; LDH: Lactate Dehydrogenase; eGFR: Estimated Glomerular Filtration Rate; CRP: C-Reactive Protein; PT: Prothrombin Time; aPTT: Activated Partial Thromboplastin Time

Characteristics	Patient 1	Patient 2	Patient 3
Age (Years)	52	74	31
Sex	Male	Female	Male
Imaging Features	CT scan: Right lung pulmonary emboli. Extensive diffuse bilateral ground-glass infiltrates.	CT: Bilateral pulmonary emboli within the main pulmonary arteries and lower lobe pulmonary arteries.	CT: Right lower lobe subsegmental pulmonary emboli. No evidence of right heart strain. No evidence of main or central pulmonary emboli.
Treatment	Enoxaparin 1 mg/kg Q12hours	Enoxaparin 1mg/kg Q12 hours	Apixaban 10 mg Q12 hours
Disease onset to thrombotic event (Days)	5	14	21
Laboratory Findings			
WBC	10,200 ul [4.8 - 10.8 k/uL}	13,100 ul [4.8 - 10.8 k/uL}	13,800 ul [4.8 - 10.8 k/uL}
Differential Count			
Total Neutrophils	7,200 ul [1.5 - 8.0 k/uL]	11,200 ul [1.5 - 8.0 k/uL]	7,960 ul [1.5 - 8.0 k/uL]
Total Lymphocytes	1,500 ul [1.0 - 4.8 k/uL]	700 ul [1.0 - 4.8 k/uL]	1200 ul [1.0 - 4.8 k/uL]
Total Monocytes	1000 ul [0.3 - 0.5 k/uL]	1,100 ul [0.3 - 0.5 k/uL]	1,500 ul [0.3 - 0.5 k/uL]
Platelet Count	185 [150 - 400 k/uL]	216 [150 - 400 k/uL]	316 [150 - 400 k/uL]
Hemoglobin	13.4 g/dL [12.0 - 16.0 g/dL]	14.9 g/dL [12.0 - 16.0 g/d	11.6 g/dL [12.0 - 16.0 g/d
Albumin	3.5 gr/dL [3.2 - 4.6 g/dL]	2.8 gr/dL [3.2 - 4.6 g/dL]	3.7 gr/dL [3.2 - 4.6 g/dL]
ALT	22 unit/L [5 - 40 unit/L]	14 unit/L [5 - 40 unit/L]	56 unit/L [5 - 40 unit/L]
AST	28 unit/L [9 - 36 unit/L]	34 unit/L [9 - 36 unit/L]	18 unit/L [9 - 36 unit/L]
LDH	687 Units / L [110 - 210 unit/L]	737 Units/L [110 - 210 unit/L]	203 Units/L [110 - 210 unit/L]
Creatinine	1.8 mg/dL [0.5 - 1.5 mg/dL]	0.5 mg/dL [0.5 - 1.5 mg/dL]	1 mg/dL [0.5 - 1.5 mg/dL]
EGFR	37.96 ml/min/1.73 m2	155 ml/min/1.73 m2	82.49 ml/min/1.73 m2
CRP	454 mg/L [< = 5.00 mg/dL]	80.7 mg/L [< = 5.00 mg/dL]	453 mg/L [< = 5.00 mg/dL]
Troponin I	66 ng/L [< = 12 ng/L ]	<12 [< = 12 ng/L ]	N/A [< = 12 ng/L]
PT	13.8 seconds [10.7 - 12.9 second (s)]	13.1 seconds [10.7 - 12.9 second (s)]	17.3 seconds [10.7 - 12.9 second (s)]
aPTT	30.9 seconds [25.1 - 36.5 second (s)]	29.1 seconds [25.1 - 36.5 second (s)]	38.6 seconds [ 25.1 - 36.5 second (s)]
D-Dimer	46698 ng/mL [0 - 230 ng/mL]	5021 ng/mL [0 - 230 ng/mL]	2134 ng/mL [0 - 230 ng/mL]
Serum Ferritin	673 ng/mL [13.0 - 150.0 ng/mL]	310.4 ng/mL [13.0 - 150.0 ng/mL]	1219 ng/mL [13.0 - 150.0 ng/mL]
Procalcitonin	0.48 ng/mL [0.02 - 100.00 ng/mL]	0.01 ng/mL [0.02 - 100.00 ng/mL]	N/A [0.02 - 100.00 ng/mL]

## Discussion

COVID-19 is an emerging disease with a significantly broad spectrum of presentation and clinical syndromes. This fascinating infectious disease has been associated with acute respiratory distress syndrome; severe metabolic syndromes, including diabetic ketoacidosis; severe thromboembolic syndrome, including microthrombi and both large vessel disease and small-vessel disease; severe acute tubular necrosis; electrolyte abnormalities, including hypernatremia and hyperkalemia; gastroenteric syndromes; neurologic syndromes; dermatologic syndromes; and cardiac events, including myocarditis and arrhythmias [8]. Severe presentation of the infection is associated with severe hypoxemia, rapid deterioration, and death [9]. The mechanisms on how this virus causes such a vast array of illnesses are not well understood. The presence of pulmonary embolism may account for one of the multiple potential causes of sudden death in patients with severe COVID-19 infection.

There is some evidence to suggest a hypercoagulable state in patients with COVID-19 infection. Blood coagulopathy is an innate immunity defense to fight infection. Erythrocytes kill pathogens by oxycytosis. Overactivation of oxycytosis can cause disseminated intravascular coagulation [10]. There is also evidence to suggest a possibility of an antiphospholipid syndrome [11] or pulmonary vasculopathy due to severe endothelial dysfunction because of COVID-19, which can result in microvascular thrombosis of the pulmonary capillary bed causing the hypercoagulable state. During the severe acute respiratory syndrome (SARS) outbreak in 2002-2003 and the H1N1 influenza pandemic in 2009, case reports described concurrent PE with viral lung infections [12-13]. Data from murine models suggest that COVID-19 may interact with urokinase to produce the hypercoagulable state observed in a SARS related acute lung injury [14].

Patients with COVID-19 tend to have high levels of inflammatory markers, including lactate dehydrogenase, ferritin, and D-dimer, which likely reflect the increased inflammatory burden of this disease [4]. In one study from China, it was noted that these patients have higher levels of D-dimer, fibrin degradation products (FDP), and fibrinogen (FIB) as compared to a control healthy population [15]. It was also pointed out in the same study that increasing values of D-dimer mirrored the gradual progression of disease severity. Hence, they concluded that routine monitoring seemed advisable in patients with COVID-19.

In our case series, we presented three cases of confirmed pulmonary embolism in patients with confirmed COVID-19 infection and pneumonia. These patients had no significant predisposing risk factors for deep vein thrombosis. There is evidence to support the idea that COVID-19 can predispose to a hypercoagulable state due to the presence of increased pro-inflammatory markers [16]. The development of pulmonary embolism further aggravates the already dire situation and increases morbidity and mortality. Early suspicion, detection, and treatment are crucial in managing these patients. Anticoagulant treatment is associated with decreased mortality in severe COVID-19 pneumonia [17].

## Conclusions

There is a dire need to increase awareness among health care providers regarding this manifestation of severe COVID-19 infections. Clinicians need to have a low threshold of suspicion of pulmonary embolism in patients with s COVID-19 infection who present with severe hypoxia and elevated pro-inflammatory markers (D-dimer). The timely initiation of therapy is crucial in decreasing the morbidity and mortality of COVID-19 infection. There is a need to do further studies to confirm the association between a COVID infection and pulmonary embolism, to better understand the pathophysiology of the disease.

## References

[1] Guan WJ, Ni ZY, Hu Y (2020). Clinical characteristics of coronavirus disease 2019 in China. N Engl J Med.

[2] Yang CL, Qiu X, Zeng YK, Jiang M, Fan HR, Zhang ZM (2020). Coronavirus disease 2019: a clinical review. Eur Rev Med Pharmacol Sci.

[3] Conti P, Ronconi G, Caraffa A, Gallenga C, Ross R, Frydas I, Kritas S (2020). Induction of pro-inflammatory cytokines (IL-1 and IL-6) and lung inflammation by coronavirus-19 (COVI-19 or SARS-CoV-2): anti-inflammatory strategies. J Biol Regul Homeost Agents.

[4] Kermali M, Khalsa RK, Pillai K, Ismail Z, Harky A ( 2020). The role of biomarkers in diagnosis of COVID-19 - a systematic review. Life Sci.

[5] Zuckier LS, Moadel RM, Haramati LB, Freeman LM (2020). Diagnostic evaluation of pulmonary embolism during the COVID-19 pandemic. J Nucl Med.

[6] Danzi GB, Loffi M, Galeazzi G, Gherbesi E (2020). Acute pulmonary embolism and COVID-19 pneumonia: a random association?. Eur Heart J.

[7] Rouhezamin MR, Haseli S (2020). Diagnosing pulmonary thromboembolism in COVID-19: a stepwise clinical and imaging approach. Acad Radiol.

[8] Liu K, Chen Y, Lin R, Han K (2020). Clinical features of COVID-19 in elderly patients: a comparison with young and middle-aged patients. J Infect.

[9] Greenland JR, Michelow MD, Wang L, London MJ (2020). COVID-19 infection: implications for perioperative and critical care physicians. Anesthesiology.

[10] Minasyan H, Flachsbart F (2019). Blood coagulation: a powerful bactericidal mechanism of human innate immunity. Int Rev Immunol.

[11] Zhang Y, Xiao M, Zhang S (2020). Coagulopathy and antiphospholipid antibodies in patients with Covid-19. N Engl J Med.

[12] Avnon LS, Munteanu D, Smoliakov A, Jotkowitz A, Barski L (2015). Thromboembolic events in patients with severe pandemic influenza A/H1N1. Eur J Intern Med.

[13] Ng KH, Wu AK, Cheng VC (2005). Pulmonary artery thrombosis in a patient with severe acute respiratory syndrome. Postgrad Med J.

[14] Gralinski LE, Bankhead A 3rd, Jeng S (2013). Mechanisms of severe acute respiratory syndrome coronavirus-induced acute lung injury. mBio.

[15] Giannis D, Ziogas IA, Gianni P (2020). Coagulation disorders in coronavirus infected patients: COVID-19, SARS-CoV-1, MERS-CoV and lessons from the past. J Clin Virol.

[16] Poggiali E, Bastoni D, Ioannilli E, Vercelli A, Magnacavallo A (2020). Deep vein thrombosis and pulmonary embolism: two complications of COVID-19 pneumonia?. Eur J Case Rep Intern Med.

[17] Tang N, Bai H, Chen X, Gong J, Li D, Sun Z (2020). Anticoagulant treatment is associated with decreased mortality in severe coronavirus disease 2019 patients with coagulopathy. J Thromb Haemost.

